# A Novel Convergent Synthesis of the Potent Antiglaucoma Agent Tafluprost

**DOI:** 10.3390/molecules22020217

**Published:** 2017-01-31

**Authors:** Małgorzata Krupa, Michał Chodyński, Anna Ostaszewska, Piotr Cmoch, Iwona Dams

**Affiliations:** 1 R & D Chemistry Department, Pharmaceutical Research Institute, Rydygiera 8, 01-793 Warsaw, Poland; m.krupa@ifarm.eu (M.K.); m.chodynski@ifarm.eu (M.C.); a.ostaszewska@ifarm.eu (A.O.); piotr.cmoch@icho.edu.pl (P.C.); 2 Institute of Organic Chemistry, Polish Academy of Sciences, Kasprzaka 42/52, 01-224 Warsaw, Poland

**Keywords:** tafluprost, prostaglandins, Corey lactone, Julia–Lythgoe olefination, fluorination

## Abstract

Tafluprost (AFP-168, **5**) is a unique 15-deoxy-15,15-difluoro-16-phenoxy prostaglandin F_2_α (PGF_2α_) analog used as an efficacious ocular hypotensive agent in the treatment of glaucoma and ocular hypertension, as monotherapy, or as adjunctive therapy to β-blockers. A novel convergent synthesis of **5** was developed employing Julia–Lythgoe olefination of the structurally advanced prostaglandin phenylsulfone **16**, also successfully applied for manufacturing of pharmaceutical grade latanoprost (**2**), travoprost (**3**) and bimatoprost (**4**), with an aldehyde ω-chain synthon **17**. The use of the same prostaglandin phenylsulfone **16**, as a starting material in parallel syntheses of all commercially available antiglaucoma PGF_2α_ analogs **2**–**5**, significantly reduces manufacturing costs resulting from its synthesis on an industrial scale and development of technological documentation. Another key aspect of the route developed is deoxydifluorination of a *trans*-13,14-en-15-one **30** with Deoxo-Fluor. Subsequent hydrolysis of protecting groups and final esterification of acid **6** yielded tafluprost (**5**). The main advantages are the preparation of high purity tafluprost (**5**) and the application of comparatively cheap reagents. The preparation and identification of two other tafluprost acid derivatives, tafluprost methyl ester (**32**) and tafluprost ethyl amide (**33**), are also described.

## 1. Introduction

**Figure 1:**
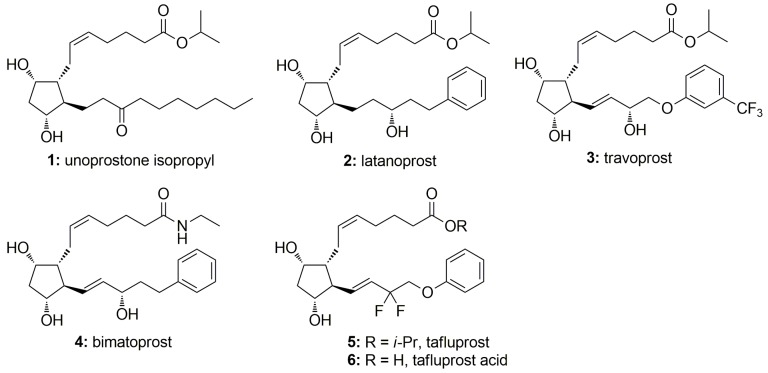
Chemical structures of PGF_2α_ analogs used in the therapy of glaucoma and ocular hypertension.

The discovery of potent and efficacious intraocular pressure (IOP) lowering PGF_2α_ analogs **1**–**5** (Figure 1) has revolutionized the treatment of ocular hypertension, a major risk factor for progression of the irreversible blinding disease glaucoma [1,2]. Until recently, free isopropyl ester prodrugs, i.e., unoprostone (**1**, Rescula), latanoprost (**2**, Xalatan) and travoprost (**3**, Travatan), and one amide prodrug bimatoprost (**4**, Lumigan) of ω-chain modified PGF_2α_ analogs have been used as first line therapy for the treatment of open angle glaucoma and ocular hypertension due to their potent IOP-lowering efficacy, low likelihood of systemic adverse effects, once-daily dosing and good patient adherence [3,4,5,6]. However, all the available commercial preparations of **1**–**4** contain preservatives to maintain the sterility of the solutions, which may impair topical tolerance of these IOP-lowering agents during long-term use [7]. New preservative-free agents with greater IOP-lowering efficacy and milder local side effects are therefore still needed.

Tafluprost (**5**) is the newest 15-deoxy-15,15-difluorinated PGF_2α_ receptor agonist possessing effective IOP-reducing effects, and also the first hypotensive drug released in a preservative-free formulation under trade names Taflotan or Saflutan in Europe and Zioptan in USA [8,9,10,11,12]. A preservative-free tafluprost formulation is as potent as a preserved one (BAK-Tapros, Osaka, Japan), but it has fewer and milder toxic effects on the eye. Therefore, it may be particularly beneficial for patients who are sensitive to preservatives, such as patients with dry eyes, and patients who discontinue medication early because of adverse events. Compared with other PGF_2α_ analogs **2**–**4**, the major modification of tafluprost is the substitution of the C-15 hydrogen and hydroxyl group with two fluorine atoms. Tafluprost is a prodrug containing an isopropyl ester moiety that is rapidly hydrolyzed by corneal esterases to the pharmacologically active metabolite tafluprost free acid (AFP-172, **6**) [13]. Tafluprost acid has 12 times the affinity for the prostaglandin F receptor as does latanoprost acid, while having almost no affinity for related receptors, such as prostaglandin D or E [14].

**Scheme 1:**
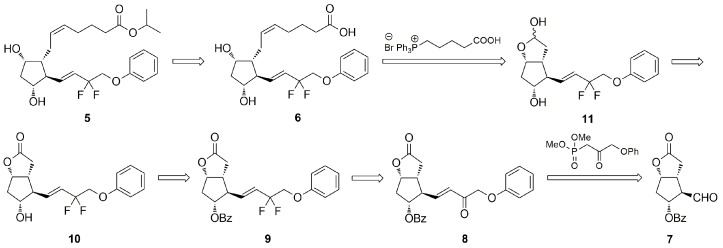
Corey strategy for synthesis of tafluprost (**5**).

PGF_2α_ analogs **1**–**5** are mostly synthesized by one of the known variants of the Corey method, in which lower and upper side chains are sequentially attached in a specific order to a derivative of the commercially available (−)-Corey aldehyde/lactone [9,15,16,17,18,19,20,21,22,23,24,25,26]. In 2004, Matsumura et al. [9] published the first synthesis of tafluprost (**5**) from Corey aldehyde derivative **7** comprising a sequence of the following reactions: (a) the installation of the lower side chain (ω-chain) via a Horner–Wadsworth–Emmons condensation of the Corey aldehyde **7** with dimethyl (2-oxo-3-phenoxypropyl)phosphonate; (b) difluorination of the ω-chain 15-one **8** with morpholinosulfur trifluoride to a 15-deoxy-15,15-difluoro derivative **9**; (c) removal of benzoyl group with potassium carbonate; (d) reduction of the lactone moiety to a lactol **11** with diisobutylaluminum hydride; (e) addition of the α-chain by Wittig olefination of **11** with an ylide prepared from 4-carboxybutyltriphenylphpsphonium bromide, leading to a *cis*-5,6-alkene; and (f) esterification of the acid **6** with isopropyl iodide in the presence of 1,8-diazabicyclo[5.4.0]undec-7-ene (Scheme 1). Thus far, however, there was no convergent methodology that would allow effective and highly diastereoselective preparation of a whole series of antiglaucoma PGF_2α_ analogs **2**–**5** from a common and structurally advanced prostaglandin intermediate.

**Scheme 2:**
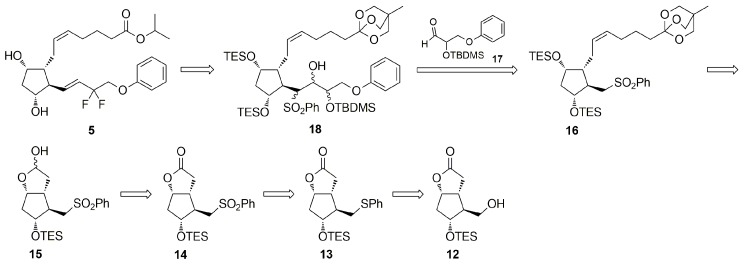
Novel strategy for convergent synthesis of tafluprost (**5**) from prostaglandin phenylsulfone **16** and aldehyde ω-chain synthon **17**, based on the reverse order of side chain attachment to the derivative of Corey lactone (**12**).

In 2007, Martynow et al. [27] reported preparation of the structurally advanced prostaglandin phenylsulfone (5*Z*)-**16** (Scheme 2) with the attached α-chain possessing a carboxyl group protected by 4-methyl-2,6,7-trioxabicyclo[2.2.2]octane (methyl-OBO). The choice of the methyl-OBO carboxyl-masking group was predicted by its high stability under basic reaction conditions commonly applied during prostaglandin synthesis. On the other side, the compounds of the 4-methyl-2,6,7-trioxabicyclo[2.2.2]octane structure are easily hydrolyzed under acidic conditions to corresponding 2,2-bis(hydroxymethyl)-1-propyl esters, which can be in turn converted into other alkyl esters, like for example alkyl esters, salts or carboxylic acids. Thus, the ω-chain elongation of the phenylsulfone **16** by S_N_2 alkylation with enantiomerically pure alkyl halides allowed the synthesis of 13,14-dihydro-15-ol PGF_2α_ analogs, such as latanoprost (**2**) [27]. The Julia–Lythgoe olefination of the sulfone **16** with enantiomerically pure aldehyde ω-chain synthons allowed the synthesis of *trans*-13,14-en-15-ol PGF_2α_ analogs, such as travoprost (**3**) and bimatoprost (**4**) [28,29]. One of the main reasons for choosing this method of phenylsulfone **16** ω-chain elongation was formation of *trans*-13,14-ene as the only product of olefination. Compared to triphenylphosphonium or phosphonate derivatives, used in competitive Wittig or Horner-Wadsworth-Emmons olefinations, phenylsulfones are mostly stable and crystal compounds that could be easily purified to pharmaceutical grade at early stage of prostaglandin synthesis. Recently, we envisaged that the prostaglandin key intermediate **16** could also find the application in the convergent synthesis of tafluprost (**5**), depending on the structure of the aldehyde ω-chain synthon and the sequence of chemical reactions needed for the ω-chain elongation and functionalization (Scheme 2). The use of the same structurally advanced prostaglandin phenylsulfone **16**, as a starting material in parallel syntheses of all commercially available antiglaucoma PGF_2α_ analogs **2**–**5**, would significantly reduce manufacturing costs resulting from its synthesis on an industrial scale and development of technological documentation. Since the orthoester **16** is stable at about 0 °C in the presence of a trace of pyridine, large amounts of **16** can be manufactured and stored in a pure form for a long time allowing the synthesis of the desired PGF_2α_ analog **2**–**5** at any time.

Glaucoma is one of the most common causes of irreversible visual impairment and blindness. It is estimated that over 60 million individuals were afflicted with open-angle and angle-closure glaucoma as of 2010, which will increase to almost 80 million by 2020 [30]. The disease affects all ethnicities, and 6.7 million people are bilaterally blind consequently. As a result of a growing demand for prostaglandin antiglaucoma drugs in the pharmaceutical market, we have developed a novel synthesis of tafluprost (**5**) from the advanced prostaglandin phenylsulfone **16** and α-hydroxy protected aldehyde **17** (Scheme 2) based on the reversed order of side chain attachment to the Corey lactone via Wittig and Julia–Lythgoe olefinations. Since the fourth commercially available PGF_2α_ drug tafluprost (**5**) contains the *trans*-13,14-en-15-deoxy-15,15-difluoro moiety in the ω-chain, when compared to latanoprost (**2**) travoprost (**3**) and bimatoprost (**4**), some modifications in an earlier developed approach for the highly diastereoselective construction of the ω-chain allylic moiety to incorporate the desired C-15 difluorinated center needed to be developed.

Most common PGF_2α_ analogs, latanoprost (**2**), travoprost (**3**), bimatoprost (**4**) and tafluprost (**5**), are licensed for the reduction of elevated intraocular pressure in patients with open angle glaucoma and ocular hypertension, but their non approved use as eyelash enhancers is becoming popular, especially in patients with eyelashes hypotrichosis [31]. There are other prostaglandin derivatives, such as tafluprost methyl ester (**32**) [32,33] and tafluprost ethyl amide (**33**) [34], that could be used in antiglaucoma ophthalmic compositions or cosmetics because they appear capable of IOP reduction and influencing eyelash growth; however, their efficacy and safety has not been fully studied and evaluated yet. Therefore, there is also a strong demand for a convenient process that is suitable for preparation of high purity tafluprost methyl ester (**32**) and tafluprost ethyl amide (**33**) on a commercial scale.

## 2. Results and Discussion

Based on our previous work on ω-chain elongation of the prostaglandin phenylsulfone **16** with the aldehyde ω-chain synthons of travoprost and bimatoprost [28,29], we envisaged that the Julia–Lythgoe olefination [35,36,37] of the sulfone **16** with the α-hydroxy protected aldehyde **17** should give a mixture of β-hydroxysulfones **18** (Scheme 2), which via reductive dehydroxy-desulfonylation and deprotection/protection of methyl-OBO and hydroxyl groups followed by oxidation of the 15-ol and deoxydifluorination of 13,14-en-15-one could enable construction of the ω-chain *trans*-13,14-en-15-deoxy-15,15-difluoro moiety. Irrespective of stereochemical configuration of the C-2 carbon atom in the aldehyde **17**, corresponding to the C-15 carbon atom in tafluprost (**5**), the deprotection of *tert*-butyldimethylsilyl protected hydroxyl group and its oxidation to a 15-one followed by its deoxydifluorination should lead to the non-chiral C-15 gem-difluorinated carbon atom in the final prostaglandin analog **5**. Therefore, for economic reasons, the 2-(*tert*-butyl-dimethylsilyloxy)-3-phenoxypropanal (**17**) used for the construction of the ω-chain of tafluprost (**5**) should be prepared as a racemate.

### 2.1. Synthesis of the Aldehyde ω-Chain Synthon ***17***

**Scheme 3:**
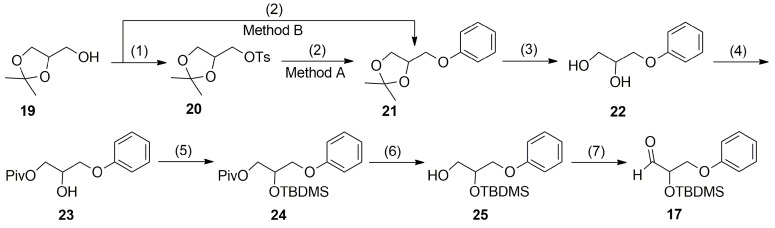
Synthesis of the racemic aldehyde ω-chain synthon **17**. *Conditions:* (1) TsCl, Py, 0 °C for 10 min, then r.t. overnight, 99% yield; (2) Method A: phenol, NaOH, EtOH-H_2_O, reflux for 30 h, 90% yield. Method B: phenol, PPh_3_, DIAD, toluene, 99–100 °C for 18 h, 96% yield; (3) 1.0 M HCl, acetone, 70 °C for 1 h, 98% yield; (4) PivCl, Py-CH_2_Cl_2_, 0 °C for 1 h, then 1.5 h at r.t., 93% yield; (5) TBDMSCl, ImH, DMF, 0 °C for 15 min, then 18 h at r.t., 94% yield; (6) DIBAL-H, CH_2_Cl_2_, −78 °C for 20 min, then 2 h at r.t., 95% yield; (7) DMP, NaHCO_3_, CH_2_Cl_2_, 0 °C for 15 min, then 1 h at r.t., 94% yield.

To the best of our knowledge, the aldehyde ω-chain synthon **17** has never been obtained in a racemic form. In 2010, Lin et al. reported the preparation of optically active (*R*)-**17** based on epoxide ring-opening of (*R*)-benzyl glicydyl ether with phenol in the presence of organic base [38]. The protection of the resulting secondary alcohol with *tert*-butyldimethylsilyl trifluoromethanesulfonate followed by removing of benzyl group and Dess-Martin oxidation of the (*S*)-**25** gave the optically active aldehyde (*R*)-**17**. Although the route described above could be applied to supply of the racemic aldehyde **17**, the cost of benzyl glicydyl ether and the use of heavy metal catalyzed hydrogenation process limit its large-scale application in the pharmaceutical industry. Therefore, a more industrially applicable procedure for synthesis of racemic 2-(*tert*-butyldimethylsilyloxy)-3-phenoxypropanal (**17**) (Scheme 3) was developed based on a synthetic route applied earlier for the preparation of the aldehyde ω-chain synthon of travoprost [28].

### 2.2. Synthesis of Tafluprost *(**5**)*, Tafluprost Methyl Ester *(**32**)* and Tafluprost Ethyl Amide *(**33**)*

**Scheme 4:**
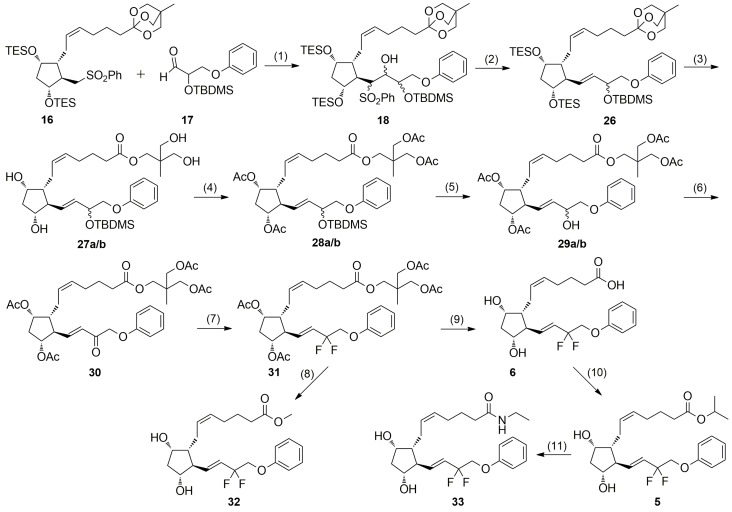
Synthesis of tafluprost (**5**), tafluprost methyl ester (**32**) and tafluprost ethylamide (**33**) from phenylsulfone **16** and aldehyde **17**. *Reagents and conditions*: (1) LDA, THF, −78 °C, 1 h; (2) Na/Hg, Na_2_HPO_4_, MeOH, 0 °C for 60 min, then 16 h at r.t.; (3) PPTS, CH_2_Cl_2_, MeOH, 3 h at r.t., 79% yield from **16**; (4) TEA, DMAP, Ac_2_O, CH_2_Cl_2_, 97% yield; (5) CAS, MeOH, CH_2_Cl_2_, 7 h at r.t., 82% yield; (6) DMP, NaHCO_3_, CH_2_Cl_2_, 99% yield; (7) Deoxo-Fluor, CH_2_Cl_2_; 0 °C for 15 min, then reflux for 24 h, 78% yield; (8) K_2_CO_3_, MeOH, 24 h at r.t., 76% yield; (9) LiOH H_2_O, MeOH, overnight, 98% yield; (10) *i*-PrI, DBU, acetone, 21 h at r.t., 83% yield; (11) EtNH_2_, H_2_O, 60 h at r.t.; 85% yield.

The efficient ω-chain elongation of phenylsulfone **16** with sterically hindered aldehyde **17** to the mixture of diastereoisomeric β-hydroxysulfones **18** was achieved by using freshly prepared LDA (Scheme 4). Since significant losses of **18** were observed during column chromatography due to limited stability of the methyl-OBO protecting group on silica gel, the crude mixture of β-hydroxysulfones **18** was subjected to reductive elimination with a fresh 20% sodium amalgam to afford the methyl-OBO protected prostaglandin precursor **26**. It is well documented that sodium amalgam reduction of aliphatic α-hydroxysulfones furnishes olefins having the trans configuration of the ω-chain 13,14-double bond [36]. The phosphate-buffered conditions of Trost [39] were used to ensure the optimal pH conditions and prevent deprotection of the cyclopentane hydroxyl groups. Similarly to the methyl-OBO protected β-hydroxysulfones **18**, the methyl-OBO protected prostaglandin intermediate **26** was not purified by means of silica gel column chromatography. Significantly, there was no need for purification of the prostaglandin intermediates **18** and **26**, which was a major advantage for any future scale-up. The triethylsilyl and methyl-OBO masking groups in prostaglandin precursor **26** were all removed under the action of pyridinium *p*-toluenesulfonate (PPTS) in a mixture of CH_2_Cl_2_-MeOH, immediately followed by esterification of free hydroxyl groups with acetic anhydride to the diastereoisomeric 2,2-bis(acetoxymethyl)propyl ester **28a**/**b**. The compound **28a**/**b** was purified by silica gel flash chromatography; however, only a sample of one of the diastereoisomers was isolated and identified by spectroscopic methods. The unique stereochemical features of the Julia–Lythgoe olefination allowed preserving the relative *cis*/*trans* stereochemistry of side chains in the isolated prostaglandin intermediate. Additionally, the appropriately functionalized aldehyde ω-chain synthon **17** with bulky *tert*-butyldimethylsilyl group at C-2, in agreement with literature data for olefination with aldehydes with steric encumbrance [40], afforded *trans*-13,14-alkenes **28a**/**b** as the only products of Julia–Lythgoe olefination. The *tert*-butyldimethylsilyl protecting group was removed with camphor-10-sulfonic acid (CSA) to afford a mixture of diastereoisomeric 15-OH prostaglandin derivatives **29a**/**b**. Similarly to the ester **28a**/**b**, silica gel flash chromatography allowed isolation and identification by spectroscopic methods of only one C-15 *epi*-isomer from the mixture of diastereoisomeric alcohols **29a**/**b**. Most importantly, since the chirality of C-15 carbon atom disappears during tafluprost (**5**) final synthesis, there was no need for purification and separation of the diastereoisomeric prostaglandin intermediates **27a**/**b**, **28a**/**b** and **29a**/**b**, which additionally was a major advantage for any future scale-up. A mixture of diastereiosmeric alcohols **29a**/**b** was then oxidized with Dess-Martin periodinane [41] to a 15-keto intermediate **30**. The use of Dess-Martin periodinane avoided some difficulties encountered with other methods, e.g., oxidation of 13,14-en-15-ols with toxic and unstable DDQ [19], such as adverse products, long reaction times, difficult workup procedures or the need to apply a large excess of the oxidizing agent. Additionally, the product of the oxidation route could be easily purified with short column chromatography. The structure of **30** was confirmed by spectroscopic methods.

Although a gem-difluoride unit has been drawn more attention recently in medicinal chemistry, there is no general method to prepare allyl difluorides from the corresponding 13,14-en-15-ones efficiently [42,43]. Therefore, we studied the reaction using some commercially available fluorinating agents, such as (diethylamino)sulfur trifluoride (DAST), (diethylamino)-difluorosulfonium tetrafluoroborate (XtalFluor-E), difluoro(morpholino)sulfonium tetrafluoro-borate (XtalFluor-M), bis(2-methoxyethyl)aminosulfur trifluoride (Deoxo-Fluor) and 4-*tert*-butyl-2,6-dimethylphenylsulfur trifluoride (Fluolead). After exploring various possibilities, Deoxo-Fluor was found to be the best reagent of choice. Thus, the fluorination reaction of 13,14-en-15-one **30** with Deoxo-Fluor in boiling CH_2_Cl_2_ for 24 h afforded geminal difluoride **31** with 78% yield.

The deprotection of acetyl and 2,2-bis(acetoxymethyl)propyl groups in **31** by means of potassium carbonate in MeOH yielded tafluprost methyl ester (**33**) in 76% yield. The ester **31** was hydrolyzed with lithium hydroxide monohydrate and, after acidification with citric acid, tafluprost acid (**6**) was isolated in almost quantitative yield. In the final step of the synthesis, acid **6** was successfully esterified with *i*-Pr/DBU in acetone to afford tafluprost (**5**). The crude **5** was purified by silica gel flash chromatography and identified by spectroscopic methods. The α-chain allylic carbons C-4 and C-7 of tafluprost are seen in the ^13^C-NMR spectrum as two singlets located at 25.7 and 26.6 ppm, which is characteristic for 5,6-*cis* diastereoisomers of PGF_2α_ analogs [27]. The fluorination of C-15 carbonyl group in **30** with Deoxo-Fluor leads to several significant effects in NMR spectra, which are important from the point of view of structure conformation/determination. The biggest change related to the replacement of oxygen with two fluorine atoms leads to strong shielding increase of C-15 nucleus by ca. 80 ppm and appearance of this signal in ^13^C-NMR spectrum as a triplet with relative large ^1^*J*(C-F) coupling of ca. 240 Hz. Similar but rather smaller effects are observed for neighboring protons and carbons. In the case of ^1^H-NMR spectrum, transition from **30** to **5** leads to shielding increase of all protons in ω-chain by ca. 0.3–0.5 ppm. The same is true for ^13^C nuclei but the shielding effects are stronger and vary in the range from 3 to 9 ppm. The biggest one (ca. 9 ppm) is observed for C-13 carbon. Two- and three- bonds C-F couplings visible in ^13^C-NMR spectrum as triplets are of typical values for such difluorine compounds containing double bond [44] and are as follows: 8.8 Hz, 25.0 Hz and 34.9 Hz for C-13, C-14 and C-16 nuclei, respectively. Complete ^1^H/^13^C and ^19^F-NMR chemical shifts assignment for tafluprost (**5**) is presented in the Experimental Section 3.6.10.

Most synthetic procedures used to attain prostamides F_2α_ proceed via prostaglandin acid and its esterification to the corresponding isopropyl ester, subsequently followed by amidation with ethylamine [24,25]. Thus, tafluprost (**5**) was successfully amidated to tafluprost ethyl amide (**33**) with ethylamine aqueous solution. The crude **33** was purified by silica gel flash chromatography and identified by spectroscopic methods.

## 3. Experimental Section

### 3.1. General Information

d,l-2,2-Dimethyl-4-(hydroxymethyl)-1,3-dioxolane (98%), phenol (99%), Dess-Martin periodinane (97%), trimethyl acetyl chloride (99%), *tert*-butyldimethylsilyl chloride (97%), imidazole (99%), diisobutylaluminum hydride solution (1.0 M in toluene), diisopropylamine (99.5%), butyllithium solution (1.6 M in hexanes), sodium mercury amalgam (99.9% trace metal basis, Na 20% beads), *p*-toluenesulfonyl chloride (98%), diisopropyl azodicarboxylate (97%), triphenylphosphine (99%), pyridinium *p*-toluenesulfonate (98%), acetic anhydride (99%), triethylamine (99%), 4-(dimethyl-amino)pyridine (99%), camphor-10-sulfonic acid (98%), bis(2-methoxyethyl)aminosulfur trifluoride solution (Deoxo-Fluor solution, 50% in THF), 1,8-diazabicyclo[5.4.0]undec-7-ene (99%), 2-iodopropane (99%), ethylamine (70% in H_2_O), pyridine (anhydrous, 99.8%), *N*,*N*-dimethylformamide (anhydrous, 99.5%), were purchased from Sigma Aldrich and Iris Biotech Gmbh chemical companies. 1-[(*Z*)-6-{(1*R*,2*R*,3*R*,5*S*)-2-[(Phenyl-sulfonyl)methyl]-3,5-bis(triethylsilyloxy)cyclopentyl}hex-4-enyl-4-methyl-2,6,7-trioxabicyclo[2.2.2]-octane [27] was manufactured by Pharmaceutical Research Institute (Warsaw, Poland). Reactions requiring anhydrous conditions were carried out using flame-dried glassware, which was cooled to 20 °C in a desiccators under an argon atmosphere. All moisture- or air-sensitive reactions were run under an argon atmosphere. Air-sensitive reagents were transferred via syringe or cannula and were introduced to the apparatus through rubber septa. Solids were introduced under a positive pressure of argon. Reactions were cooled via external cooling baths: ice water (0 °C) or dry-acetone (−78 °C). Heating was accomplished by either a heating mantle or silicone oil bath. Deionized water was used for all aqueous extractions and for obtaining all aqueous solutions. Solvents were removed under reduced pressure using standard rotary evaporators. The course of all reactions and the purity of products were checked by thin-layer chromatography (TLC). Analytical TLC was performed on silica gel DC-Alufolien Kieselgel 60 F_254_ (Merck, Darmstadt, Germany) with mixtures of hexanes, ethyl acetate, dichloromethane, methanol, acetonitrile and toluene in various ratios as developing systems. Compounds were detected by spraying the plates with 1% Ce(SO_4_)_2_/2% H_3_[P(Mo_3_O_10_)_4_] in 10% H_2_SO_4_ followed by heating to 120 °C. Preparative column chromatography was carried out on silica gel (Kieselgel 60, 40–63 μm, 230–400 mesh, Merck) with mixtures of ethyl acetate, hexanes, dichloromethane, methanol, *tert*-butyl-methyl ether in varying ratios as eluents. Flash chromatography was run with a positive air pressure generated by handheld, pear-shaped rubber pumps.

### 3.2. Optical Rotation

Optical rotations were measured with a Perkin Elmer 341 automatic polarimeter in Me_2_CO or CH_2_Cl_2_ solutions as the solvents with percent concentrations.

### 3.3. FT-IR Spectroscopy

IR spectra were taken for liquid films or KBr pellets on a Nicolet Impact 410 FT-IR spectrophotometer.

### 3.4. NMR Spectroscopy

The NMR spectra of all the compounds were measured in CDCl_3_ solutions with a Varian VNMRS-600 (600 MHz) or Varian VNMRS-500 (500 MHz) at temperature 298 K using TMS (for ^1^H/^13^C-NMR measurements) or CCl_3_F (for ^19^F-NMR measurements) as internal standards. Concentration of all solutions used for measurements was about 20–30 mg of compounds in 0.6 mL of solvent.

### 3.5. Mass Spectrometry

HRMS spectra were recorded on an AMD 604 Inectra Gmbh spectrometer or a Mariner PE Biosystem ESI-TOF spectrometer.

### 3.6. Syntheses

The synthesis of the aldehyde ω-chain synthon **17** from the commercially available d,l-1,2-isopropylidene glycerol (**19**) is shown in Scheme 3. The detailed descriptions of the preparation of compounds **20**–**25** and **17** as well as their physical and spectroscopic data are provided in the Supplementary Materials.

#### 3.6.1. 1-{(*Z*)-6-[(1*R*,2*R*,3*R*,5*S*)-2-[(1*R*/1*S*,2*R*/2*S*,3*R*/3*S*)-3-(*tert*-Butyldimethylsilyloxy)-4-phenoxy-1-(phenylsulfonyl)butyl]-3,5-bis(triethylsilyloxy)cyklopentyl]hex-4-enyl}-4-methyl-2,6,7-trioxabicyclo-[2.2.2]octane (**18**)

*n*-BuLi (28.6 mL, 45.8 mmol, 1.6 M in hexanes) was added dropwise to a solution of diisopropylamine (7.96 mL, 45.8 mmol) in anhydrous THF (12 mL) at −78 °C under an argon atmosphere. After 15 min at −78 °C, the phenylsulfone **16** (21.0 g, 30.2 mmol) in anhydrous THF (40 mL) was added dropwise with vigorous stirring. The reaction mixture was stirred at −60 °C for 30 min and the aldehyde **17** (14.0 g, 50.0 mmol) in anhydrous THF (10 mL) was added dropwise. After being stirred at −60 °C for another 30 min, TLC analysis (6% MeCN/toluene) indicated disappearance of the starting phenylsulfone **16**. The cold reaction was quenched with saturated aqueous NaCl solution (30 mL). The resulting layers were separated and the aqueous phase was extracted with THF (3 × 25 mL). The combined organic extracts were washed with brine (150 mL), dried over Na_2_SO_4_, filtered and concentrated in vacuo. The crude product was carefully dried in vacuo to yield a mixture of diastereoisomeric hydroxysulfones **18** (38.2 g), as a yellow oil. The hydroxysulfones **18** were directly used for the next step without further purification.

#### 3.6.2. 1-{(4*Z*)-6-[(1*R*,2*R*,3*R*,5*S*)-2-[(1*E*,3*R*/3*S*)-3-(*tert*-Butyldimethylsilyloxy)-4-(phenoxy)but-1-enyl]-3,5-bis(triethylsilyloxy)cyclopentyl]hex-4-enyl}-4-methyl-2,6,7-trioxabicyclo[2.2.2] octane (**26**)

The crude mixture of diastereoisomeric hydroxysulfones **18** (38.2 g) was dissolved in THF (25 mL) and saturated methanolic solution of Na_2_HPO_4_ (120 mL) was added in one portion. On cooling to 0 °C under an argon atmosphere, sodium amalgam (20%, 25.0 g, 217.4 mmol Na) was added portionwise over 60 min with vigorous stirring. The cooling bath was removed and the resulting suspension was stirred at room temperature for 16 h to disappearance of the starting hydroxysulfones **18** (TLC, MeCN/toluene 1:1). The solution was decanted from the remaining amalgam and concentrated under reduced pressure. The residue was diluted with H_2_O (60 mL) and AcOEt (60 mL). The resulting layers were separated and the aqueous phase was extracted with AcOEt (3 × 50 mL). The combined organic extracts were washed with brine (150 mL) and dried over anhydrous Na_2_SO_4_. Filtration and evaporation in vacuo furnished the crude product **26** (38.1 g) as a light yellow oil. The crude olefin **26** was directly used for the next step without further purification.

#### 3.6.3. 2,2-Bis(hydroxymethyl)propyl (5*Z*)-7-{(1*R*,2*R*,3*R*,5*S*)-2-[(1*E*,*3R*/3*S*)-3-(*tert*-butyldimethylsilylo-xy)-4-(phenoxy)but-1-enyl]-3,5-dihydroxycyclopentyl}hept-5-enoate (**27a**/**b**)

PPTS (28.0 g, 111.42 mmol) was added to a solution of the crude silyl protected prostaglandin derivatives **26** (38.1 g) in a mixture of CH_2_Cl_2_-MeOH (1:1, 300 mL). The resulting brown solution was stirred for 3 h, whereupon brine (150 mL) and saturated aqueous solution NaHCO_3_ (150 mL) were added. After being stirred for 15 min, the organic solvents were evaporated under reduced pressure. The residue was extracted with AcOEt (3 × 100 mL). The combined organic extracts were dried over anhydrous Na_2_SO_4_. Filtration and evaporation in vacuo furnished the crude tetraol (24.8 g). Purification by silica gel flash chromatography with gradient elution 1%–6% MeOH-CH_2_Cl_2_ afforded a sample of prostaglandin analog (+)-**27a** (84 mg) and an inseparable mixture of diastereoisomeric tetraols **27a**/**b** (14.5 g, 79% yield from phenylsulfone **16**) as a light yellow viscous oil. (+)-**27a**: *R*_f_ = 0.42 (50% CH_3_CN/toluene). ${\left[\alpha \right]}_{D}^{20}$ = +17.72 (*c* 1.0, CH_2_Cl_2_). FT-IR (thin film) ν (cm^−1^): 3393, 2946, 2929, 2899, 2857, 1731, 1717, 1600, 1587, 1497, 1471, 1388, 1301, 1248, 1172, 1143, 1044, 974, 837, 810, 779, 754, 733, 692, 596, 509. ^1^H-NMR (CDCl_3_, 600 MHz) δ (ppm): 0.10 (two s, 6H, (CH_3_)_2_Si), 0.84 (s, 3H, (-C(CH_3_)(CH_2_OH)_2_), 0.91 (s, 9H, (CH_3_)_3_C-Si), 1.57 (m, 1H, cyclopentane CH-1), 1.71 (m, 2H, α-chain CH_2_-3), 1.81 (m, 1H, one of the cyclopentane CH_2_-4 group), 2.05–2.20 (m, 4H, α-chain CH_2_-4, one of the α-chain CH_2_-7 group, one of the cyclopentane CH_2_-4 group), 2.32 (m, 1H, one of the α-chain CH_2_-7 group), 2.33–2.40 (m, 3H, α-chain CH_2_-2 and cyclopentane CH-2), 3.52 (d, *J* = 11.5 Hz, 2H, -CH_2_OH), 3.57 (d, *J* = 11.5 Hz, 2H, -CH_2_OH), 3.86 (d, *J* = 6.0 Hz, 2H, ω-chain CH_2_-4), 3.99 (m, 1H, cyclopentane CH-3), 4.15–4.20 (m, 3H, -OCH_2_C(CH_3_)(CH_2_OH)_2_ and cyclopentane CH-5), 4.51 (m, 1H, ω-chain CH-3), 5.36 (m, 1H, α-chain CH-5), 5.42 (m, 1H, α-chain CH-6), 5.62–5.68 (m, 2H, ω-chain CH-1 and CH-2), 6.88 (m, 2H, aromatic CH-2 and CH-6), 6.93 (m, 1H, aromatic CH-4), 7.27 (m, 2H, aromatic CH-3 and CH-5). ^13^C-NMR (150 MHz, CDCl_3_) δ (ppm): −4.7 (CH_3_-Si), −4.6 (CH_3_-Si), 16.9 (-C(CH_3_)(CH_2_OH)_2_), 18.4 ((CH_3_)_3_C-Si), 24.8 (α-chain C-3), 25.8 (3C, (CH_3_)_3_C-Si), 26.1 (α-chain C-7), 26.6 (α-chain C-4), 33.6 (α-chain C-2), 40.6 (-C(CH_3_)(CH_2_OH)_2_), 42.8 (cyclopentane C-4), 50.6 (cyclopentane C-1), 56.0 (cyclopentane C-2), 66.5 (-OCH_2_C(CH_3_)(CH_2_OH)_2_), 67.5 (2C, -OCH_2_C(CH_3_)(CH_2_OH)_2_), 71.6 (ω-chain C-3), 72.2 (ω-chain C-4), 73.6 (cyclopentane C-5), 78.4 (cyclopentane C-3), 114.4 (2C, aromatic C-2 and C-6), 120.7 (aromatic C-4), 129.3 (α-chain CH-6), 129.4 (2C, aromatic C-3 and C-5), 129.5 (α-chain CH-5), 131.0 (ω-chain C-2), 133.0 (ω-chain C-1), 158.8 (aromatic C-1), 174.7 (C=O). HRMS (ESI): calcd. for C_33_H_54_O_8_NaSi [M + Na]^+^ 629.3486; found 629.3474.

#### 3.6.4. 2,2-Bis(acetoxymethyl)propyl (5*Z*)-7-{(1*R*,2*R*,3*R*,5*S*)-3,5-diacetoxy-2-[(1*E*,3*R*/3*S*)-3-(*tert*-butyldi-methylsilyloxy)-4-(phenoxy)but-1-enyl]cyclopentyl}hept-5-enoate (**28a**/**b**)

TEA (23.7 mL, 173.3 mmol), DMAP (0.2 g) and Ac_2_O (8.2 mL, 86.5 mol) were added to a solution of the diastereoisomeric prostaglandin derivatives **27a**/**b** (10.5 g, 17.3 mmol) in CH_2_Cl_2_ (100 mL). The mixture was stirred for 1 h at room temperature prior the addition of saturated aqueous solution NH_4_Cl (100 mL). The resulting layers were separated and the aqueous phase was extracted with CH_2_Cl_2_ (3 × 50 mL). The combined organic extracts were washed with saturated aqueous solution NaHCO_3_ (50 mL) and dried over anhydrous MgSO_4_. Filtration and evaporation in vacuo furnished the crude product, which was purified by silica gel flash chromatography with gradient elution 5%–10% AcOEt-hexanes to give a sample of ester (+)-**28a** (70 mg) and an inseparable mixture of diastereoisomeric esters **28a**/**b** (12.97 g, yield 97% yield). (+)-**28a**: *R*_f_ = 0.35 (25% AcOEt/hexanes). ${\left[\alpha \right]}_{D}^{20}$ = +23.72 (*c* 1.0, CH_2_Cl_2_). FT-IR (thin film) ν (cm^−1^): 2954, 2932, 2896, 2857, 1740, 1717, 1600, 1587, 1497, 1472, 1375, 1240, 1173, 1150, 1043, 974, 917, 838, 811, 779, 756, 692, 605, 510. ^1^H-NMR (CDCl_3_, 600 MHz) δ (ppm): 0.08 (two s, 6H, (CH_3_)_2_Si), 0.89 (s, 9H, (CH_3_)_3_C-Si), 1.00 (s, 3H, (-C(CH_3_)(CH_2_OH)_2_), 1.64 (m, 3H, one of the cyclopentane CH_2_-4 group and α-chain CH_2_-3), 1.68 (m, 1H, cyclopentane CH-1), 1.95–2.16 (three s and two m, 16H, four -COOCH_3_ groups, α-chain CH_2_-4 and α-chain CH_2_-7), 2.28 (t, *J* = 7.7 Hz, 2H, α-chain CH_2_-2), 2.55–2.65 (m, 2H, one proton of the cyclopentane CH_2_-4 group and cyclopentane CH-2), 3.83 (dd, *J* = 9.4 and 5.2 Hz, 1H, one of the ω-chain CH_2_-4 group), 3.89 (dd, *J* = 9.4 and 6.9 Hz, 1H, one of the ω-chain CH_2_-4 group), 3.99 (s, 6H, -OCH_2_C(CH_3_)(CH_2_OH)_2_), 4.50 (m, 1H, ω-chain CH-3), 4.90 (m, 1H, cyclopentane CH-3), 5.09 (m, 1H, cyclopentane CH-5), 5.30–5.38 (m, 2H, α-chain CH-5 and α-chain CH-6), 5.62 (dd, *J* = 8.3 and 15.3 Hz, 1H, ω-chain CH-1), 5.67 (dd, *J* = 5.6 and 15.3 Hz, 1H, ω-chain CH-2), 6.87 (m, 2H, aromatic CH-2 and CH-6), 6.93 (m, 1H, aromatic CH-4), 7.27 (m, 2H, aromatic CH-3 and CH-5). ^13^C-NMR (150 MHz, CDCl_3_) δ (ppm): −4.7 (CH_3_-Si), −4.6 (CH_3_-Si), 17.0 (-C(CH_3_)(CH_2_OH)_2_), 18.3 ((CH_3_)_3_C-Si), 20.7, 21.0 and 21.2 (4C, four -COOCH_3_ groups), 24.7 (α-chain C-3), 24.9 (α-chain C-7), 25.8 (3C, (CH_3_)_3_C-Si), 26.6 (α-chain C-4), 33.5 (α-chain C-2), 38.2 (–C(CH_3_)(CH_2_OH)_2_), 39.0 (cyclopentane C-4), 47.3 (cyclopentane C-1), 51.8 (cyclopentane C-2), 65.6 (-OCH_2_C(CH_3_)(CH_2_OH)_2_), 65.7 (2C, -OCH_2_C(CH_3_)(CH_2_OH)_2_), 71.7 (ω-chain C-3), 72.1 (ω-chain C-4), 74.3 (cyclopentane C-5), 77.8 (cyclopentane C-3), 114.4 (2C, aromatic C-2 and C-6), 120.9 (aromatic C-4), 128.0 (α-chain CH-6), 129.4 (2C, aromatic C-3 and C-5), 129.7 (α-chain CH-5), 131.4 (ω-chain C-1), 133.2 (ω-chain C-2), 158.8 (aromatic C-1), 170.4, 170.6 and 170.7 (4C, four –COOCH_3_ groups). 173.1 (-CH_2_COOCH_2_-). HRMS (ESI): calcd. for C_41_H_62_O_12_NaSi [M + Na]^+^ 797.3908; found 797.3898.

#### 3.6.5. 2,2-Bis(acetoxymethyl)propyl (5*Z*)-7-{(1*R*,2*R*,3*R*,5*S*)-3,5-Diacetoxy-2-[(1*E*,3*R*/3*S*)-4-phenoxy-3-hydroxybut-1-enyl]cyclopentyl}hept-5-enoate (**29a**/**b**)

Camphorosulfonic acid (1.12 g, 4.81 mmol) was added to a solution of diastereoisomeric esters **28a**/**b** (11.3 g, 14.58 mmol) in a mixture of MeOH-CH_2_Cl_2_ (1:1, 100 mL). The resulting solution was stirred for 7 h, whereupon NaHCO_3_ (0.47 g) was added. After being stirred for 30 min, the mixture was filtered through a Büchner funnel and the filtrate was concentrated under reduced pressure. The crude product was purified by silica gel flash chromatography with gradient elution 5%–40% AcOEt-hexanes to give a sample of alcohol (+)-**29a** (59 mg) and an inseparable mixture of diastereoisomeric alcohols **29a**/**b** (6.94 g, 82% yield). (+)-**29a**: *R*_f_ = 0.37 (50% AcOEt/hexanes). ${\left[\alpha \right]}_{D}^{20}$ = +32.46 (*c* 1.0, MeOH). FT-IR (thin film) ν (cm^−1^): 3501, 2940, 1740, 1600, 1497, 1378, 1243, 1173, 1152, 1042, 975, 915, 814, 757, 693, 606, 512. ^1^H-NMR (CDCl_3_, 600 MHz) δ (ppm): 1.00 (s, 3H, (-C(CH_3_)(CH_2_OH)_2_), 1.60–1.74 (m, 4H, one of the cyclopentane CH_2_-4 group, cyclopentane CH-1, α-chain CH_2_-3), 1.95–2.10 (three s and two m, 14H, four -COOCH_3_ groups and α-chain CH_2_-4), 2.14 (m, 2H, α-chain CH_2_-7), 2.30 (t, *J* = 7.7 Hz, 2H, α-chain CH_2_-2), 2.55 (m, 1H, one of the cyclopentane CH_2_-4 group), 2.62 (m, 1H, cyclopentane CH-2), 3.91 (dd, *J* = 7.2 and 9.0 Hz, 1H, one of the α-chain CH_2_-4), 3.99 (m, 7H, -OCH_2_C(CH_3_)(CH_2_OH)_2_ and one of the ω-chain CH_2_-4 group), 4.54 (m, 1H, ω-chain CH-3), 4.94 (m, 1H, cyclopentane CH-3), 5.11 (m, 1H, cyclopentane CH-5), 5.30–5.38 (m, 2H, α-chain CH-5 and α-chain CH-6), 5.71 (dd, *J* = 5.4 and 15.6 Hz, 1H, ω-chain CH-1), 5.74 (dd, *J* = 7.2 and 15.6 Hz, 1H, ω-chain CH-2), 6.92 (m, 2H, aromatic CH-2 and CH-6), 6.98 (m, 1H, aromatic CH-4), 7.29 (m, 2H, aromatic CH-3 and CH-5). ^13^C-NMR (150 MHz, CDCl_3_) δ (ppm): 17.0 (-C(CH_3_)(CH_2_OH)_2_), 20.7, 21.1 and 21.2 (4C, four -COOCH_3_ groups), 24.6 (α-chain C-3), 24.8 (α-chain C-7), 26.6 (α-chain C-4), 33.5 (α-chain C-2), 38.2 (–C(CH_3_)(CH_2_OH)_2_), 39.0 (cyclopentane C-4), 47.3 (cyclopentane C-1), 52.0 (cyclopentane C-2), 65.6 (-OCH_2_C(CH_3_)(CH_2_OH)_2_), 65.7 (2C, -OCH_2_C(CH_3_)(CH_2_OH)_2_), 70.5 (ω-chain C-3), 71.7 (ω-chain C-4), 74.3 (cyclopentane C-5), 77.8 (cyclopentane C-3), 114.6 (2C, aromatic C-2 and C-6), 121.2 (aromatic C-4), 128.1 (α-chain CH-6), 129.5 (2C, aromatic C-3 and C-5), 129.8 (α-chain CH-5), 131.1 (ω-chain C-2), 132.6 (ω-chain C-1), 158.5 (aromatic C-1), 170.4, 170.6 and 170.7 (4C, four -COOCH_3_ groups). 173.2 (-CH_2_COOCH_2_-). HRMS (ESI): calcd. for C_35_H_48_O_12_Na [M + Na]^+^ 683.3043; found 683.3040.

#### 3.6.6. 2,2-Bis(acetoxymethyl)propyl (5*Z*)-7-{(1*R*,2*R*,3*R*,5*S*)-3,5-Diacetoxy-2-[(1*E*)-4-(phenoxy)-3-oxo-but-1-enyl]cyclopentyl}hept-5-enoate (**30**)

Dess-Martin periodinane (4.48 g, 10.56 mmol) was added portionwise to a suspension of diastereoisomeric alcohols **29a**/**b** (5.8 g, 8.8 mmol) and dry NaHCO_3_ (2.22 g, 26.4 mmol) in anhydrous CH_2_Cl_2_ (50 mL). After being stirred for 1 h at room temperature, TLC analysis (AcOEt/hexanes, 1:1) indicated disappearance of the starting alcohols **29a**/**b**. Saturated aqueous NaHCO_3_ (100 mL) and Na_2_SO_3_ (8.87 g, 70.4 mmol) were then added simultaneously and the mixture was stirred at room temperature for 30 min The resulting layers were separated and the aqueous phase was extracted with CH_2_Cl_2_ (3 × 50 mL). The combined extracts were washed with water brine (100 mL) and dried over Na_2_SO_4_. Filtration and evaporation in vacuo furnished the crude product as a light yellow oil, which was purified by flash column chromatography (silica gel, 2%–20% AcOEt/hexanes) to give the ketone **30** (5.76 g, 99% yield) as a colorless oil. *R*_f_ = 0.54 (50% AcOEt/hexanes). ${\left[\alpha \right]}_{D}^{20}$ = +40.67 (*c* 1.0, CH_2_Cl_2_). FT-IR (thin film) ν (cm^−1^): 2944, 1740, 1697, 1626, 1600, 1589, 1496, 1474, 1435, 1376, 1239, 1174, 1043, 984, 917, 785, 757, 693, 645, 606, 509. ^1^H-NMR (CDCl_3_, 600 MHz) δ (ppm): 1.01 (s, 3H, -C(CH_3_)(CH_2_OH)_2_), 1.64 (m, 2H, α-chain CH_2_-3), 1.73 (ddd, *J* = 1.3, 4.2 and 15.8 Hz, 1H, one of the cyclopentane CH_2_-4 group), 1.84 (m, 1H, cyclopentane CH-1), 1.92–2.10 (three s and two m, 15H, four -COOCH_3_ groups, α-chain CH_2_-4 and one of the α-chain CH_2_-7 group), 2.15 (m, 1H, one of the α-chain CH_2_-7 group), 2.29 (t, *J* = 7.6 Hz, 2H, α-chain CH_2_-2 group), 2.58 (ddd, *J* = 5.6, 9.1 and 15.8 Hz, 1H, one of the cyclopentane CH_2_-4 group), 2.76 (m, 1H, cyclopentane CH-2 group), 3.99 (m, 6H, -OCH_2_C(CH_3_)(CH_2_OH)_2_), 4.72 (two d, *J* = 16.4 Hz, ω-chain CH_2_-4), 4.98 (ddd, *J* = 4.2, 7.4 and 11.7 Hz, 1H, cyclopentane CH-3), 5.12 (m, 1H, cyclopentane CH-5), 5.29 (m, 1H, α-chain CH-6), 5.34 (m, 1H, α-chain CH-5), 6.51 (dd, *J* = 0.8 and 15.7 Hz, 1H, ω-chain CH-2), 6.90 (m, 2H, aromatic CH-2 and CH-6), 6.92 (dd, *J* = 9.1 and 15.7 Hz, 1H, ω-chain CH-1), 6.99 (m, 1H, aromatic CH-4), 7.29 (m, 2H, aromatic CH-3 and CH-5). ^13^C-NMR (150 MHz, CDCl_3_) δ (ppm): 17.0 (-C(CH_3_)(CH_2_OH)_2_), 20.7, 21.1 and 21.2 (4C, four -COOCH_3_ groups), 24.6 (α-chain C-3), 25.0 (α-chain C-7), 26.6 (α-chain C-4), 33.5 (α-chain C-2), 38.2 (-C(CH_3_)(CH_2_OH)_2_), 39.1 (cyclopentane C-4), 47.7 (cyclopentane C-1), 52.4 (cyclopentane C-2), 65.6 (-OCH_2_C(CH_3_)(CH_2_OH)_2_), 65.7 (2C, -OCH_2_C(CH_3_)(CH_2_OH)_2_), 72.0 (ω-chain C-4), 74.3 (cylopentane C-5), 77.2 (cyclopentane C-3), 114.6 (2C, aromatic C-2 and C-6), 121.7 (aromatic C-4), 127.2 (ω-chain CH-2), 127.4 (α-chain CH-6), 129.6 (2C, aromatic C-3 and C-5), 130.3 (α-chain CH-5), 147.7 (ω-chain C-1), 157.8 (aromatic C-1), 170.2, 170.4 and 170.7 (4C, four -COOCH_3_ groups), 173.1 (-CH_2_COOCH_2_), 195.5 (ω-chain C-3). HRMS (ESI): calcd. for C_35_H_46_O_12_Na [M + Na]^+^ 681.2887; found 681.2877.

#### 3.6.7. 2,2-Bis(acetoxymethyl)propyl (5*Z*)-7-{(1*R*,2*R*,3*R*,5*S*)-3,5-diacetoxy-2-[(1*E*)-3,3-difluoro-4-(phe-noxy)-but-1-enyl]cyclopentyl}hept-5-enoate (**31**)

Deoxo-Fluor (39.1 mL, 91.2 mmol, 50% in THF) was added dropwise to a stirred solution of ketone **30** (5.0 g, 7.6 mmol) in CH_2_Cl_2_ (60 mL) at 0 °C under an argon atmosphere. The resulting mixture was refluxed for 24 h, cooled to 0–5 °C, whereupon saturated aqueous solution of NaHCO_3_ (200 mL) was added dropwise. After being stirred for 30 min at room temperature, the layers were separated and the aqueous phase was extracted with CH_2_Cl_2_ (3 × 50 mL). The combined extracts were washed with brine (100 mL) and dried over Na_2_SO_4_. Filtration and evaporation in vacuo furnished the crude product as a light yellow oil, which was purified by flash column chromatography (silica gel, 5%–20% AcOEt/hexanes) to give the difluoro derivative **31** (4.03 g, 78% yield) as a colorless oil. *R*_f_ = 0.64 (50% AcOEt/hexanes). ${\left[\alpha \right]}_{D}^{20}$ = +27.75 (*c* 1.0, MeOH). FT-IR (thin film) ν (cm^−1^): 2945, 1739, 1599, 1496, 1378, 1239, 1159, 1046, 977, 849, 757, 693, 606, 509. ^1^H-NMR (CDCl_3_, 600 MHz) δ (ppm): 1.00 (s, 3H, -C(CH_3_)(CH_2_OH)_2_), 1.64 (m, 2H, α-chain CH_2_-3), 1.70 (ddd, *J* = 1.3, 4.5 and 15.8 Hz, 1H, one proton of the cyclopentane CH_2_-4 group), 1.77 (m, 1H, cyclopentane CH-1), 1.95–2.10 (three s and two m, 14H, four -COOCH_3_ groups and α-chain CH_2_-4), 2.14 (m, 2H, α-chain CH_2_-7), 2.28 (t, *J* = 7.6 Hz, 2H, α-chain CH_2_-2), 2.58 (ddd, *J* = 5.8, 9.0 and 15.8 Hz, 1H, one proton of the cyclopentane CH_2_-4), 2.68 (m, 1H, cyclopentane CH-2), 4.00 (m, 6H, -OCH_2_C(CH_3_)(CH_2_OH)_2_), 4.19 (m, 2H, ω-chain CH_2_-4), 4.95 (ddd, *J* = 4.5, 7.7 and 11.9 Hz, 1H, cyclopentane CH-3), 5.12 (m, 1H, cyclopentane CH-5), 5.32–5.40 (m, 2H, α-chain CH-5 and α-chain CH-6), 5.84 (m, 1H, ω-chain CH-2), 6.11 (m, 1H, ω-chain CH-1), 6.91 (m, 2H, aromatic CH-2 and CH-6), 7.00 (m, 1H, aromatic CH-4), 7.30 (m, 2H, aromatic CH-3 and CH-5). ^13^C-NMR (150 MHz, CDCl_3_) δ (ppm): 17.0 (-C(CH_3_)(CH_2_OH)_2_), 20.8, 21.1 and 21.2 (4C, four -COOCH_3_ groups), 24.6 (α-chain C-3), 24.8 (α-chain C-7), 26.6 (α-chain C-4), 33.5 (α-chain C-2), 38.2 (-C(CH_3_)(CH_2_OH)_2_), 39.0 (cyclopentane C-4), 47.4 (cyclopentane C-1), 51.8 (cyclopentane C-2), 65.6 (-OCH_2_C(CH_3_)(CH_2_OH)_2_), 65.8 (2C, -OCH_2_C(CH_3_)(CH_2_OH)_2_), 69.5 (^2^*J*_C-F_ = 35.0 Hz, ω-chain C-4), 74.2 (cyclopentane C-5), 77.2 (cyclopentane C-3), 114.7 (2C, aromatic C-2 and C-6), 118.0 (^1^*J*_C-F_ = 240.5 Hz, ω-chain C-3), 121.8 (aromatic C-4), 125.2 (^2^*J*_C-F_ = 25.0 Hz, ω-chain C-2), 127.6 (α-chain CH-6), 129.6 (2C, aromatic C-3 and C-5), 130.1 (α-chain CH-5), 136.8 (^3^*J*_C-F_ = 8.8 Hz, ω-chain C-1), 157.9 (aromatic C-1), 170.3, 170.6 and 170.8 (4C, four -COOCH_3_ groups), 173.1 (-CH_2_COOCH_2_). HRMS (ESI): calcd. for C_35_H_46_O_11_NaF_2_ [M + Na]^+^ 703.2906; found 703.2900.

#### 3.6.8. Methyl (5*Z*)-7-{(1*R*,2*R*,3*R*,5*S*)-2-[(1*E*)-3,3-difluoro-4-(phenoxy)but-1-enyl]-3,5-dihydroxycyclo-pentyl}hept-5-enoate (**32**)

K_2_CO_3_ (0.46 g, 3.3 mmol) was added to a solution of ester **31** (0.75 g, 1.1 mmol) in MeOH (25 mL). The resulting suspension was stirred for 24 h at room temperature and then concentrated under reduced pressure. The residue was diluted with H_2_O (50 mL) and the product was extracted with AcOEt (3 × 25 mL). The combined extracts were washed with brine (100 mL) and dried over Na_2_SO_4_. Filtration and evaporation in vacuo furnished the crude product as a light yellow oil, which was purified by flash column chromatography (silica gel, 10%–40% AcOEt/hexanes) to give the methyl ester **32** (0.35 g, 76% yield) as a colorless oil. *R*_f_ = 0.48 (75% AcOEt/hexanes). ${\left[\alpha \right]}_{D}^{20}$ = +23.97 (*c* 1.0, MeOH). FT-IR (thin film) ν (cm^−1^): 3412, 3009, 2935, 1733, 1676, 1599, 1497, 1456, 1438, 1303, 1250, 1155, 1055, 975, 935, 848, 756, 692, 509. ^1^H-NMR (CDCl_3_, 600 MHz) δ (ppm): 1.62 (m, 1H, cyclopentane CH-1), 1.68 (m, 2H, α-chain CH_2_-3), 1.85 (m, 1H, one proton of the cyclopentane CH_2_-4 group), 2.07 (m, 1H, one of the α-chain CH_2_-4 group), 2.10–2.18 (m, 3H, one of the α-chain CH_2_-4 group, one of the α-chain CH_2_-7 group and one of the cyclopentane CH_2_-4 group), 2.18–2.26 (br s, 2H, two -OH), 2.27–2.36 (m, 3H, one of the α-chain CH_2_-7 group and α-chain CH_2_-2), 2.47 (m, 1H, cyclopentane CH-2), 3.66 (s, 3H, -COOCH_3_), 4.03 (m, 1H, cyclopentane CH-3), 4.17–4.23 (m, 2H, cyclopentane CH-5 and α-chain CH_2_-4), 5.33–5.42 (m, 2H, α-chain CH-5 and α-chain CH-6), 5.80 (m, 1H, ω-chain CH-2), 6.11 (m, 1H, ω-chain CH-1), 6.91 (m, 2H, aromatic CH-2 and CH-6), 7.00 (m, 1H, aromatic CH-4), 7.30 (m, 2H, aromatic CH-3 and CH-5). ^13^C-NMR (150 MHz, CDCl_3_) δ (ppm): 24.7 (α-chain C-3), 25.7 (α-chain C-7), 26.6 (α-chain C-4), 33.3 (α-chain C-2), 43.0 (cyclopentane C-4), 50.5 (cyclopentane C-1), 51.6 (-COOCH_3_), 55.8 (cyclopentane C-2), 69.5 (^2^*J*_C-F_ = 35.0 Hz, ω-chain C-4), 73.3 (cyclopentane C-5), 78.0 (cyclopentane C-3), 114.7 (2C, aromatic C-2 and C-6), 118.1 (^1^*J*_C-F_ = 241.0 Hz, ω-chain C-3), 121.8 (aromatic C-4), 123.6 (^2^*J*_C-F_ = 24.9 Hz, ω-chain C-2), 128.6 (α-chain CH-6), 129.6 (2C, aromatic C-3 and C-5), 130.0 (α-chain CH-5), 138.6 (^3^*J*_C-F_ = 9.1 Hz, ω-chain C-1), 157.9 (aromatic C-1), 174.3 (C=O). HRMS (ESI): calcd. for C_23_H_30_O_5_NaF_2_ [M + Na]^+^ 447.1959; found 447.1960.

#### 3.6.9. (5*Z*)-7-{(1*R*,2*R*,3*R*,5*S*)-2-[(1*E*)-3,3-difluoro-4-(phenoxy)but-1-enyl]-3,5-dihydroxycyclopentyl}-hept-5-enoic acid (**6**)

LiOH·H_2_O (1.4 g, 33.36 mmol) was added in one portion to a solution of ester **31** (2.84 g, 4.17 mmol) in MeOH (50 mL). The resulting suspension was stirred overnight resulting in disappearance of the starting material **31** (TLC, MeOH/AcOEt 1:6). After cooling and evaporating the solvent, the residual solid was dissolved in water (150 mL) and washed with Et_2_O (25 mL) to remove organic impurities. The water layer was acidified with citric acid to pH 4–5 and the product was extracted with AcOEt (3 × 50 mL). The combined organic extracts were dried over anhydrous Na_2_SO_4_, filtered and evaporated in vacuo to afford the crude acid. Purification by silica gel flash chromatography with gradient elution 10%–50% MeOH/AcOEt afforded the pure tafluprost acid (**6**) (1.69 g, 98% yield) as a thick pale yellow oil. *R*_f_ = 0.49 (15% MeOH/AcOEt). ${\left[\alpha \right]}_{D}^{20}$ = +17.98 (*c* 1.0, MeOH). FT-IR (thin film) ν (cm^−1^): 3370, 2933, 1709, 1599, 1497, 1457, 1406, 1302, 1249, 1155, 1055, 974, 934, 849, 755, 692, 509. ^1^H-NMR (CDCl_3_, 600 MHz) δ (ppm): 1.59 (m, 1H, cyclopentane CH-1), 1.66 (m, 2H, α-chain CH_2_-3), 1.84 (m, 1H, one of the cyclopentane CH_2_-4), 2.00–2.20 (m, 3H, one of the α-chain CH_2_-4, one of the α-chain CH_2_-7 and one of the cyclopentane CH_2_-4 group), 2.28–2.36 (m, 3H, one of the α-chain CH_2_-7 and α-chain CH_2_-2), 2.47 (m, 1H, cyclopentane CH-2), 4.03 (m, 1H, cyclopentane CH-3), 4.17–4.22 (m, 3H, cyclopentane CH-5 and ω-chain CH_2_-4), 5.33–5.42 (m, 2H, α-chain CH-5 and α-chain CH-6), 5.80 (m, 1H, ω-chain CH-2), 6.10 (m, 1H, ω-chain CH-1), 6.91 (m, 2H, aromatic CH-2 and CH-6), 6.99 (m, 1H, aromatic CH-4), 7.29 (m, 2H, aromatic CH-3 and CH-5). ^13^C-NMR (150 MHz, CDCl_3_) δ (ppm): 24.5 (α-chain C-3), 25.7 (α-chain C-7), 26.4 (α-chain C-4), 33.2 (α-chain C-2), 42.8 (cyclopentane C-4), 50.4 (cyclopentane C-1), 55.5 (cyclopentane C-2), 69.5 (^2^*J*_C-F_ = 34.7 Hz, ω-chain C-4), 73.4 (cyclopentane C-5), 77.8 (cyclopentane C-3), 114.8 (2C, aromatic C-2 and C-6), 118.2 (^1^*J*_C-F_ = 240.0 Hz, ω-chain C-3), 121.8 (aromatic C-4), 123.7 (^2^*J*_C-F_ = 25.0 Hz, ω-chain C-2), 128.8 (α-chain CH-6), 129.6 (2C, aromatic C-3 and C-5), 129.9 (α-chain CH-5), 138.6 (^3^*J*_C-F_ = 8.7 Hz, ω-chain C-1), 158.0 (aromatic C-1), 178.8 (C=O). HRMS (ESI): calcd. for C_22_H_28_O_5_NaF_2_ [M + Na]^+^ 433.1803; found 433.1801.

#### 3.6.10. Isopropyl (5*Z*)-7-{(1*R*,2*R*,3*R*,5*S*)-2-[(1*E*)-3,3-difluoro-4-(phenoxy)but-1-enyl]-3,5-dihydroxycy-clopentyl}hept-5-enoate (**5**)

DBU (3.4 mL, 22.82 mmol) was added dropwise to a stirred solution of acid **6** (1.34 g, 3.26 mmol) in anhydrous Me_2_CO (20 mL) at 0 °C under an argon atmosphere. The mixture was allowed to warm to room temperature, whereupon 2-iodopropane (2.3 mL, 22.82 mmol) was added dropwise. The resulting solution was stirred for 21 h resulting in disappearance of the starting acid **6** (TLC, MeOH/AcOEt 1:6). The reaction was quenched with AcOEt (100 mL). The resulting white solid was filtered off and washed with AcOEt (3 × 15 mL). The filtrate and washings were combined and acidified with 3% citric acid solution to pH 5–6. The resulting layers were separated and the aqueous phase was extracted with AcOEt (3 × 15 mL). The combined extracts were washed with saturated NaHCO_3_ (100 mL), brine (100 mL) and dried over anhydrous Na_2_SO_4_. Filtration and evaporation in vacuo furnished the crude product, which was purified by silica gel flash chromatography with gradient elution 10%–45% AcOEt/hexanes to afford the title compound **5** (1.23 g, 83% yield) as a pale yellow oil. This sample was further purified by preparative HPLC on silica gel to give pharmaceutical grade tafluprost (**5**) as a thick colorless oil. ${\left[\alpha \right]}_{D}^{20}$ = +22.46 (*c* 1.0, MeOH). Lit. ${\left[\alpha \right]}_{D}^{23}$ = +21.6 (*c* 1.0, CHCl_3_) [45]. *R*_f_ = 0.48 (75% AcOEt/hexanes). FT-IR (thin film) ν (cm^−1^): 3408, 2980, 2935, 1727, 1599, 1497, 1456, 1375, 1303, 1250, 1154, 1108, 1055, 973, 936, 848, 821, 755, 691, 509. ^1^H-NMR (CDCl_3_, 600 MHz) δ (ppm): 1.22 (d, 6H, -CH(CH_3_)_2_), 1.61 (m, 1H, cyclopentane CH-1), 1.67 (m, 2H, α-chain CH_2_-3), 1.85 (m, 1H, one of the cyclopentane CH_2_-4 group), 2.06 (m, 1H, one of the cyclopentane CH_2_-4 group), 2.09–2.16 (m, 3H, one of the α-chain CH_2_-4, one of the α-chain CH_2_-7 group and one of the cyclopentane CH_2_-4 group), 2.24–2.36 (m, 3H, α-chain CH_2_-2 and one of the α-chain CH_2_-7), 2.47 (m, 1H, cyclopentane CH-2), 4.02 (m, 1H, cyclopentane CH-3), 4.17–4.22 (m, 2H, cyclopentane CH-5 and ω-chain CH_2_-4), 5.00 (sept, *J* = 6.30 Hz, 1H, -CH(CH_3_)_2_), 5.33–5.42 (m, 2H, α-chain CH-5 and α-chain CH-6), 5.80 (m, 1H, ω-chain CH-2), 6.10 (m, 1H, ω-chain CH-1), 6.92 (m, 2H, aromatic CH-2 and CH-6), 7.00 (m, 1H, aromatic CH-4), 7.30 (m, 2H, aromatic CH-3 and CH-5). ^13^C-NMR (150 MHz, CDCl_3_) δ (ppm): 21.8 (2s, 2C, -CH(CH_3_)_2_), 24.8 (α-chain C-3), 25.7 (α-chain C-7), 26.6 (α-chain C-4), 34.0 (α-chain C-2), 42.9 (cyclopentane C-4), 50.5 (cyclopentane C-1), 55.7 (cyclopentane C-2), 67.7 (-CH(CH_3_)_2_), 69.5 (^2^*J*_C-F_ = 34.9 Hz, ω-chain C-4), 73.3 (cyclopentane C-5), 77.9 (cyclopentane C-3), 114.8 (2C, aromatic C-2 and C-6), 118.2 (^1^*J*_C-F_ = 240.0 Hz, ω-chain C-3), 121.8 (aromatic C-4), 123.6 (^2^*J*_C-F_ = 25.0 Hz, ω-chain C-2), 128.6 (α-chain CH-6), 129.6 (2C, aromatic C-3 and C-5), 130.1 (α-chain CH-5), 138.6 (^3^*J*_C-F_ = 8.8 Hz, ω-chain C-1), 157.9 (aromatic C-1), 173.5 (C=O). ^19^F-NMR (470 MHz, CDCl_3_) δ (ppm): −102.6 (^2^*J*_F-F_ = 255.8 Hz), −103.1 (^2^*J*_F-F_ = 255.8 Hz). HRMS (ESI): calcd. for C_25_H_34_O_5_NaF_2_ [M + Na]^+^ 475.2272; found 475.2272.

#### 3.6.11. (5*Z*)-*N*-Ethyl-7-{(1*R*,2*R*,3*R*,5*S*)-2-[(1*E*)-3,3-difluoro-4-(phnoxy)but-1-enyl]-3,5-dihydroxycy-clopentyl}hept-5-enamide (**33**)

EtNH_2_ (50 mL, 629.0 mmol, 70% in water) was added in one portion to the tafluprost **5** (0.8 g, 1.77 mmol). The resulting solution was stirred for 60 h to disappearance of the starting material **5** (TLC, 7% MeOH/CH_2_Cl_2_). The excess of EtNH_2_ was then removed by evaporation under reduced pressure and the aqueous residue was diluted with brine (50 mL) and AcOEt (50 mL). The resulting layers were separated and the aqueous phase was extracted with AcOEt (3 × 25 mL). The combined extracts were dried over anhydrous Na_2_SO_4_. Filtration and evaporation in vacuo furnished the crude product, which was purified by silica gel flash chromatography with gradient elution from 1%–5% MeOH/CH_2_Cl_2_ to afford the title compound **33** (0.66 g, 85% yield) as colorless oil. *R*_f_ = 0.34 (7% MeOH/CH_2_Cl_2_). ${\left[\alpha \right]}_{D}^{20}$ = +27.55 (*c* 1.0, MeOH). FT-IR (thin film) ν (cm^−1^): 3308, 3095, 2934, 1645, 1599, 1553, 1497, 1456, 1294, 1249, 1155, 1055, 974, 933, 848, 755, 691, 509. ^1^H-NMR (CDCl_3_, 600 MHz) δ (ppm): 1.12 (t, 3H, -CH_2_CH_3_), 1.58 (m, 1H, cyclopentane CH-1), 1.67 (m, 2H, α-chain CH_2_-3), 1.87 (m, 1H, one of the cyclopentane CH_2_-4 group), 2.00 (m, 1H, one of the α-chain CH_2_-4 group), 2.05–2.22 (m, 5H, one of the α-chain CH_2_-4 group, one of the α-chain CH_2_-7 group, one of the cyclopentane CH_2_-4 group and α-chain CH_2_-2), 2.38 (m, 1H, one of the α-chain CH_2_-7 group), 2.49 (m, 1H, cyclopentane CH-2), 2.94 (br s, 2H, two -OH), 3.26 (m, 2H, -CH_2_CH_3_), 4.04 (m, 1H, cyclopentane CH-3), 4.16–4.22 (m, 2H, cyclopentane CH-5 and ω-chain CH_2_-4), 5.33–5.42 (m, 2H, α-chain CH-5 and α-chain CH-6), 5.69 (br s, 1H, -NH-), 5.79 (m, 1H, ω-chain CH-2), 6.11 (m, 1H, ω-chain CH-1), 6.91 (m, 2H, aromatic CH-2 and CH-6), 7.00 (m, 1H, aromatic CH-4), 7.30 (m, 2H, aromatic CH-3 and CH-5). ^13^C-NMR (150 MHz, CDCl_3_) δ (ppm): 14.8 (-CH_2_CH_3_), 25.6 (α-chain C-3), 25.7 (α-chain C-7), 26.6 (α-chain C-4), 34.4 (-CH_2_CH_3_), 35.8 (α-chain C-2), 43.1 (cyclopentane C-4), 50.7 (cyclopentane C-1), 55.7 (cyclopentane C-2), 69.5 (^2^*J*_C-F_ = 34.7 Hz, ω-chain C-4), 73.1 (cyclopentane C-5), 78.0 (cyclopentane C-3), 114.8 (2C, aromatic C-2 and C-6), 118.2 (^1^*J*_C-F_ = 240.0 Hz, ω-chain C-3), 121.8 (aromatic C-4), 123.3 (^2^*J*_C-F_ = 24.8 Hz, ω-chain C-2), 128.9 (α-chain CH-6), 129.6 (2C, aromatic C-3 and C-5), 130.0 (α-chain CH-5), 138.9 (^3^*J*_C-F_ = 8.8 Hz, ω-chain C-1), 158.0 (aromatic C-1), 173.2 (C=O). ^19^F-NMR (470 MHz, CDCl_3_) δ (ppm): −102.4 (^2^*J*_F-F_ = 255.8 Hz), −103.1 (^2^*J*_F-F_ = 255.8 Hz). HRMS (ESI): calcd. for C_24_H_33_NO_4_NaF_2_ [M + Na]^+^ 460.2275; found 460.2274.

## 4. Conclusions

Tafluprost (**5**) is the newest 15-deoxy-15,15-difluorinated PGF_2α_ receptor agonist used as an efficacious ocular hypotensive agent in the treatment of glaucoma and ocular hypertension, and also the first hypotensive drug released in a preservative-free formulation with fewer and milder toxic effects on the eye. A novel convergent synthesis of tafluprost (**5**) was developed employing Julia–Lythgoe olefination of the structurally advanced phenylsulfone (5*Z*)-**16**, also successfully applied for manufacturing of pharmaceutical grade latanoprost (**2**), travoprost (**3**) and bimatoprost (**4**), with an aldehyde ω-chain synthon **17**. The use of the same prostaglandin sulfone **16**, as a starting material in parallel syntheses of all commercially available antiglaucoma PGF_2α_ analogs **2**–**5**, significantly reduces manufacturing costs resulting from its synthesis on an industrial scale and development of technological documentation. The preparation and identification of two other tafluprost acid (**6**) derivatives, tafluprost methyl ester (**32**) and tafluprost ethyl amide (**33**), are also described.

## Supplementary Materials

Supplementary materials can be accessed at: http://www.mdpi.com/1420-3049/22/2/217/s1.
