# Supplementary material for: A Novel Convergent Synthesis of the Potent Antiglaucoma Agent Tafluprost
Source: Molecules. 2017 Jan 31;22(2):217. doi: 10.3390/molecules22020217 (PMC6155834; doi:10.3390/molecules22020217)
Supplement: Supplementary file 1 [file molecules-22-00217-s001.pdf]

Supplementary Materials: A Novel Convergent Synthesis of the Potent Antiglaucoma Agent Tafluprost

Małgorzata Krupa, Michał Chodyński, Anna Ostaszewska, Piotr Cmoch and Iwona Dams

Table of Contents **Page**

Synthesis of 2,2-dimethyl-4-(toluenesulfonyloxymethyl)-1,3-dioxolane (**20**) S1
Synthesis of 2,2-dimethyl-4-(phenoxy)methyl-1,3-dioxolane (**21**) S2
Synthesis of 3-(phenoxy)propane-1,2-diol (**22**) S2
Synthesis of 2-hydroxy-3-(phenoxy)propyl pivalate (**23**) S3
Synthesis of 2-(*tert*-butyldimethylsilyloxy)-3-(phenoxy)propyl pivalate (**24**) S3
Synthesis of 2-(*tert*-butyldimethylsilyloxy)-3-(phenoxy)propan-1-ol (**25**) S3
Synthesis of 2-(*tert*-butyldimethylosilyloxy)-3-phenoxypropanal (**17**) S4
Abbreviations S4
References S5

Synthesis of the Aldehyde ω-Chain Synthon 17

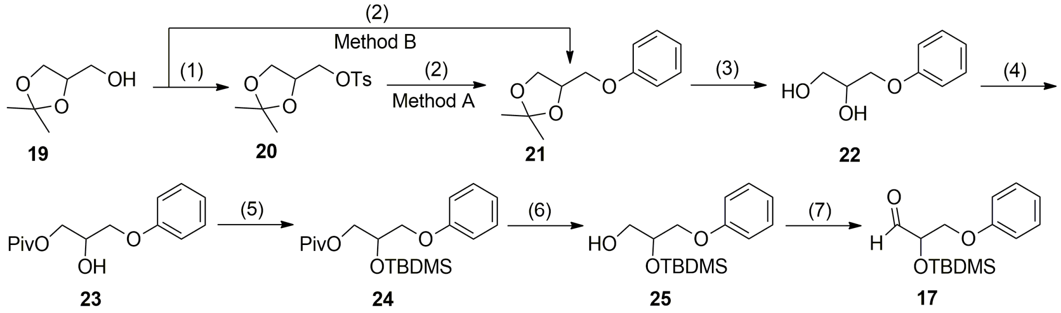

Scheme S1. Synthesis of the racemic aldehyde ω-chain synthon **17**. *Conditions:* (1) TsCl, Py, 0 °C for 10 min., then r.t. overnight, 99% yield; (2) Method A: phenol, NaOH, EtOH-H_2O, reflux for 30 h, 90% yield. Method B: phenol, PPh_3, DIAD, toluene, 99–100 °C for 18h, 96% yield; (3) 1.0 M HCl, acetone, 70 °C for 1 h, 98% yield; (4) PivCl, Py-CH_2Cl_2, 0 °C for 1 h, then 1.5 h at r.t., 93% yield; (5) TBDMSCl, ImH, DMF, 0 °C for 15 min, then 18 h at r.t., 94% yield; (6) DIBAL-H, CH_2Cl_2, −78 °C for 20 min., then 2 h at r.t., 95% yield; (7) DMP, $NaHCO_3$, CH_2Cl_2, 0 °C for 15 min., then 1h at r.t., 94% yield.

2,2-Dimethyl-4-(toluenesulfonyloxymethyl)-1,3-dioxolane (20)

p-Toluenesulfonyl chloride (31.74 g, 166.46 mol) was added portionwise over a period of 10 min. to a solution 2,2-dimethyl-4-(hydroxymethyl)-1,3-dioxolane (**19**) (20.0 g, 151.33 mmol) in anhydrous pyridine (40 mL) in an ice bath. The resulting solution was slowly brought to room temperature and stirred overnight. During that time, a white precipitate formed. The pyridine was removed under reduced pressure and the residue was diluted with AcOEt (100 mL), washed subsequently with cold aqueous 1 M HCl (2 × 150 mL), saturated $NaHCO_3$ (100 mL) and brine (200 mL). The organic layer was dried over Na_2SO_4, filtered and concentrated to give a light yellow oil. The crude product was purified by column chromatography over silica gel with gradient elution 10%–30% AcOEt/hexanes to afford 3-tosyloxy-1,2-propanediol acetonide **20** (43.16 g, 99% yield). R_f = 0.68 (1% MeOH/CH_2Cl_2). M.p. 50–51 °C (lit. m.p. 49–50 °C [1]) FT-IR (KBr) ν (cm^{-1}): 3073, 2987, 2937, 2891, 1598, 1495, 1455, 1368, 1257, 1213, 1190, 1177, 1096, 1055, 979, 829, 788, 665, 555. ^{1}H-NMR (600 MHz, $CDCl_3$) δ (ppm): 1.31 (s, 3H, CH_3-2), 1.34 (s, 3H, CH_3-2), 2.45 (s, 3H, ArC$\underline{H}_3$), 3.76 (dd, *J* = 5.1 and 8.8 Hz, 1H, one of the CH_2-5 group), 3.98 (dd, *J* = 6.0 and 10.2 Hz, 1H, one of the CH_2-1′ group), 4.01 (dd, *J* = 5.6 and 10.3 Hz, 1H, one of the CH_2-1′ group), 4.03 (dd, *J* = 6.2 and 8.8 Hz, 1H, one of the CH_2-5 group), 4.28 (m, 1H, CH-4), 7.35 (m, 2H, aromatic H-3 and H-5), 7.79 (m, 2H, aromatic H-2 and

H-6). ^{13}C-NMR (150 MHz, $CDCl_3$) δ (ppm): 21.54 (Ar-$\underline{C}H_3$), 25.05 (CH_3-2), 26.53 (CH_3-2), 66.05 (C-5), 69.44 (C-1′), 72.82 (C-4), 109.93 (C-2), 127.88 (2C, aromatic C-2 and C-6), 129.83 (2C, aromatic C-3 and C-5), 132.55 (aromatic C-1), 144.99 (aromatic C-4). HRMS (ESI): calcd. for $C_{13}H_{18}O_5NaS$ [M + Na]$^+$ 309.07672; found 309.0762.

2,2-Dimethyl-4-(phenoxy)methyl-1,3-dioxolane (21)

Method A. Sodium hydroxide (8.8 g, 220.02 mmol) was added portionwise to a stirred solution of phenol (20.71 g, 220.02 mmol) in a mixture of EtOH and H_2O (3:1, 80 mL). After being stirred for 10 min, a solution of solketal tosylate **20** (42.0 g, 146.68 mmol) in EtOH (50 mL) was added dropwise and the reaction mixture was heated at reflux for 20 h with disappearance of the starting tosylate **20** (TLC, CH_2Cl_2). The EtOH was then evaporated, the residue was treated with 10% aq NaOH (90 mL) and extracted with CH_2Cl_2 (3 × 50 mL). The combined organic layers were washed with H_2O (3 × 100 mL), dried over Na_2SO_4, filtered and evaporated to give a light yellow oil. The crude product was purified by column chromatography over silica gel with gradient elution 1%–3% AcOEt/hexanes to afford the acetonide **21** (27.37 g, 89% yield). R_f = 0.28 (CH_2Cl_2). M.p. 63–64 °C (lit. m.p. 63 °C [2]). FT-IR (KBr) ν (cm^{-1}): 3055, 3040, 2991, 2925, 2877, 1601, 1586, 1500, 1469, 1454, 1381, 1368, 1233, 1292, 1248, 1207, 1178, 1151, 1078, 1047, 1029, 1002, 973, 897, 885, 850, 838, 815, 753, 691, 613, 584, 509. ^{1}H-NMR (600 MHz, $CDCl_3$) δ (ppm): 1.40 (s, 3H, CH_3-2), 1.47 (s, 3H, CH_3-2), 3.90 (dd, *J* = 5.9 and 8.5 Hz, 1H, one of the CH_2-5 group), 3.94 (dd, *J* = 6.0 and 9.5 Hz, 1H, one of the CH_2-1′ group), 4.06 (dd, *J* = 5.5 and 9.5 Hz, 1H, one of the CH_2-1′ group), 4.17 (dd, *J* = 6.5 and 8.5 Hz, 1H, one of the CH_2-5 group), 4.48 (m, 1H, CH-4), 6.91 (m, 2H, aromatic H-2 and H-6), 6.96 (m, 1H, aromatic H-4), 7.28 (m, 2H, aromatic H-3 and H-5). ^{13}C-NMR (150 MHz, $CDCl_3$) δ (ppm): 25.4 (CH_3-2), 26.8 (CH_3-2), 66.9 (C-5), 68.7 (C-1′), 74.0 (C-4), 109.7 (C-2), 114.5 (2C, aromatic C-2 and C-6), 121.1 (aromatic C-4), 129.5 (2C, aromatic C-3 and C-5), 158.5 (aromatic C-1). HRMS (EI): calcd. for $C_{12}H_{16}O_3$ 208.1099; found 208.1109.

Method B. A solution of solketal **19** (16.85 g, 127.51 mmol) and DIAD (31.4 mL, 159.39 mmol) in anhydrous toluene (50 mL) was slowly added to a mixture of phenol (10.0 g, 106.26 mmol) and PPh_3 (41.92 g, 159.39 mmol) in anhydrous toluene (150 mL) at 90 °C over 30 min. After heating at 100 °C for another 18 h, TLC analysis (CH_2Cl_2) indicated disappearance of the starting solketal **19**. The excess of toluene (100 mL) was evaporated and the residue was put into refrigerator for several hours. Triphenylphosphine oxide was removed by filtration on a Büchner funnel and washed with cold toluene (3 × 50 mL). The filtrate and washings were combined and washed with aqueous 10% NaOH (100 mL) and H_2O (200 mL). The organic layer was dried over Na_2SO_4, filtered and concentrated to give an orange-yellow oil. The crude product was purified by column chromatography over silica gel with gradient elution 1%–3% AcOEt/hexanes to afford the acetonide **21** (21.24 g, 96% yield). M.p. 63–64 °C (lit. m.p. 63 °C [2]). The characterization data from IR and NMR spectra were identical in all aspects with those of **2** obtained according to the Method A.

3-(Phenoxy)propane-1,2-diol (22)

1.0 M aq HCl (190 mL) was added in one portion to a solution of acetonide **21** (26.0 g, 124.85 mmol) in acetone (250 mL). After heating at 70 °C for 1 h, TLC analysis (CH_2Cl_2/MeOH, 20:1) indicated the reaction was complete. The solution was cooled, acetone was then evaporated and the aqueous acidic residue was slowly neutralized with slightly more than the equivalent amount of solid $NaHCO_3$. The resulting solution was extracted with CH_2Cl_2 (4 × 50 mL). The combined extracts were washed with H_2O (3 × 100 mL), dried over Na_2SO_4, filtered and concentrated to give a light yellow oil. Purification by silica gel flash chromatography with 1%–8% MeOH/CH_2Cl_2 elution afforded the diol **22** (20.58 g, 98% yield) as a colourless oil. R_f = 0.24 (5% MeOH/CH_2Cl_2). M.p. 57–58 °C (lit. m.p. 58–59 °C [3]). FT-IR (KBr) ν (cm^{-1}): 3368, 3282, 3060, 2952, 2934, 2897, 2540, 2096, 1956, 1936, 1867, 1850, 1786, 1717, 1603, 1588, 1502, 1489, 1466, 1457, 1368, 1340, 1296, 1240, 1184, 1156, 1129, 1106, 1087, 1064, 1053, 1023, 989, 929, 895, 887, 840, 822, 811, 758, 689, 586, 510, 421. ^{1}H-NMR (600 MHz, $CDCl_3$, 25 °C) δ (ppm): 2.83 (br s, 2H, two -OH groups), 3.73 (dd, *J* = 5.8 and 11.5 Hz, 1H,

one of the CH_2-1 group), 3.82 (dd, *J* = 3.7 and 11.5 Hz, 1H, one of the CH_2-1 group), 4.01 (m, 2H, CH_2-3), 4.10 (m, 1H, CH_2-2), 6.90 (m, 2H, aromatic H-2 and H-6), 6.97 (m, 1H, aromatic H-4), 7.28 (m, 2H, aromatic H-3 and H-5). ^{13}C-NMR (150 MHz, $CDCl_3$) δ (ppm): 63.7 (C-1), 69.0 (C-3), 70.5 (C-2), 114.5 (2C, aromatic C-2 and C-6), 121.3 (aromatic C-4), 129.6 (2C, aromatic C-3 and C-5), 158.4 (aromatic C-1). HRMS (EI): calcd. for $C_9H_{12}O_3$ 168.0786; found 168.0778.

2-Hydroxy-3-(phenoxy)propyl pivalate (23)

Trimethylacetyl chloride (14.6 mL, 118.62 mmol) was added to a stirred solution of diol **22** (19.0 g, 112.97 mmol) in a mixture of CH_2Cl_2 and pyridine (1:1, 120 mL) at 0 °C under an argon atmosphere. After stirring at 0 °C for 1 h and at room temperature for 1.5 h, the reaction was quenched with crushed ice (60 g) and the solution was partitioned between CH_2Cl_2 (200 mL) and 10% aqueous HCl (300 mL). The resulting layers were separated and the aqueous phase was extracted with CH_2Cl_2 (3 × 50 mL). The combined organic extracts were washed successively with H_2O (250 mL), saturated aqueous $NaHCO_3$ (250 mL), brine (250 mL) and dried over anhydrous Na_2SO_4. Filtration and evaporation in vacuo furnished the crude ester, which was purified by flash column chromatography over silica gel with gradient elution 5%–10% AcOEt/hexanes to afford the pivalate **23** [4] (26.56 g, 93% yield) as a colourless oil. R_f = 0.41 (40% AcOEt/hexanes). FT-IR (thin film) ν (cm^{-1}): 3469, 3064, 3041, 2973, 2935, 2874, 1731, 1600, 1588, 1497, 1481, 1460, 1399, 1367, 1287, 1246, 1165, 1079, 1047, 1047, 996, 939, 884, 815, 754, 692, 590, 509. ^{1}H-NMR (600 MHz, $CDCl_3$) δ (ppm): 1.22 (s, 9H, -C(CH_3)$_3$), 2.61 (br s, 1H, -OH), 4.02 (dd, *J* = 5.7 and 9.5 Hz, 1H, one of the CH_2-3 group), 4.04 (dd, *J* = 4.5 and 9.5 Hz, 1H, one of the CH_2-3 group), 4.23 (m, 1H, CH-2), 4.28 (m, 2H, CH_2-1), 6.91 (m, 2H, aromatic H-2 and H-6), 6.98 (1H, m, aromatic H-4), 7.29 (m, 2H, aromatic H-3 and H-5). ^{13}C-NMR (150 MHz, $CDCl_3$) δ (ppm): 27.2 (3C, -C($\underline{C}H_3$)$_3$), 38.8 (-$\underline{C}$(CH_3)$_3$), 65.3 (C-1), 68.6 (C-3), 68.7 (C-2), 114.5 (2C, aromatic C-2 and C-6), 121.3 (aromatic C-4), 129.6 (2C, aromatic C-3 and C-5), 158.3 (aromatic C-1), 178.8 (C=O). HRMS (EI): calcd. for $C_{14}H_{20}O_4$ 252.1362; found 252.1357.

2-(*tert*-Butyldimethylsilyloxy)-3-(phenoxy)propyl pivalate (24)

tert-Butyldimethylsilyl chloride (18.50 g, 122.71 mmol) was added in one portion to a stirred solution of alcohol **23** (25.8 g, 102.26 mmol) and imidazole (9.05 g, 132.94 mmol) in anhydrous DMF (130 mL) at 0 °C under an argon atmosphere. The reaction was allowed to proceed for 18 h at room temperature and then quenched with crushed ice (50 g). The resulting mixture was partitioned between hexanes (100 mL) and H_2O (200 mL). The aqueous layer was extracted with hexanes (3 × 50 mL). The combined organic extracts were washed successively with H_2O (250 mL), brine (250 mL) and dried over Na_2SO_4. Filtration and evaporation in vacuo furnished the crude product as a light yellow oil, which was purified by flash column chromatography (silica gel, 1%–2% AcOEt/hexanes) to give *tert*-butyldimethylsilyl ether **24** (35.27 g, 94% yield) as a colourless oil. R_f = 0.5 (10% AcOEt/hexanes). FT-IR (thin film) ν (cm^{-1}): 3042, 2957, 2931, 2885, 2858, 1733, 1601, 1588, 1497, 1481, 1472, 1463, 1398, 1363, 1337, 1283, 1248, 1162, 1141, 1079, 1052, 1003, 979, 938, 879, 837, 811, 778, 753, 691, 589, 508. ^{1}H-NMR (600 MHz, $CDCl_3$) δ (ppm): 0.12 (s, 3H, CH_3-Si), 0.13 (s, 3H, CH_3-Si), 0.90 (s, 9H, (CH_3)$_3$C-Si), 1.22 (s, 9H, (CH_3)$_3$C-), 3.92 (dd, *J* = 6.0 and 9.3 Hz, 1H, one of the CH_2-3 group), 3.97 (dd, *J* = 4.7 and 9.3 Hz, 1H, one of the CH_2-3 group), 4.10 (m, 1H, one of the CH_2-1 group), 4.23 (m, 2H, one of the CH_2-1 group and CH-2), 6.89 (m, 2H, aromatic H-2 and H-6), 6.95 (m, 1H, aromatic H-4), 7.28 (m, 2H, aromatic H-3 and H-5). ^{13}C-NMR (150 MHz, $CDCl_3$) δ (ppm): −4.9 (CH_3-Si), −4.6 (CH_3-Si), 18.1 ((CH_3)$_3$$\underline{C}$-Si), 25.7 (3C, ($\underline{C}H_3$)$_3$C-Si), 27.2 (3C, ($\underline{C}H_3$)$_3$C-), 38.8 (($CH_3$)$_3$$\underline{C}$-), 65.7 (C-1), 69.2 (C-2), 69.4 (C-3), 114.4 (2C, aromatic C-2 and C-6), 120.9 (aromatic C-4), 129.5 (2C, aromatic C-3 and C-5), 158.6 (aromatic C-1), 178.3 (C=O). HRMS (ESI): calcd. for $C_{20}H_{34}O_4NaSi$ $[M + Na]^+$ 389.2124; found 389.2127.

2-(*tert*-Butyldimethylsilyloxy)-3-(phenoxy)propan-1-ol (25)

Diisobutylaluminum hydride (1.0 M in toluene, 236.0 mL, 236.0 mmol) was added dropwise over 20 min to a stirred solution of pivalate **24** (34.6 g, 94.39 mmol) in anhydrous THF (300 mL) at −78 °C under an argon atmosphere. The resulting mixture was allowed to warm to −20 °C for a

30 min period and stirred at this temperature for another 2 h. TLC analysis (AcOEt/hexanes, 1:9) indicated disappearance of the starting pivalate **24**. The clear colourless solution was re-cooled to −78 °C and the excess of DIBAL-H was quenched by addition of MeOH (120 mL) dropwise. On warming to 0 °C, 10% aqueous potassium sodium tartrate (250 mL) was added and the mixture was stirred vigorously at room temperature for 2 h. The resulting layers were separated and the aqueous phase was extracted with CH_2Cl_2 (3 × 75 mL). The combined extracts were washed with water (200 mL), brine (200 mL) and dried over anhydrous Na_2SO_4. Filtration and evaporation in vacuo furnished the crude product, which was purified by flash column chromatography (silica gel, 2%–10% AcOEt/hexanes) to afford the primary alcohol **25** [5] (25.33 g, 95% yield) as a colourless oil. R_f = 0.27 (20% AcOEt/hexanes). FTIR (thin film) ν (cm^{-1}): 3431, 3065, 3041, 2954, 2929, 2885, 2857, 1601, 1588, 1497, 1472, 1463, 1389, 1361, 1336, 1301, 1247, 1173, 1132, 1080, 1049, 999, 939, 880, 837, 808, 779, 753, 691, 596, 509. ^{1}H-NMR (600 MHz, $CDCl_3$) δ (ppm): 0.13 (s, 3H, CH_3-Si), 0.15 (s, 3H, CH_3-Si), 0.92 (s, 9H, $(CH_3)_3$C-Si), 3.68 (dd, *J* = 4.5 and 11.4 Hz, 1H, one of the CH_2-1 group), 3.74 (dd, *J* = 4.0 and 11.4 Hz, 1H, one of the CH_2-1 group), 3.93 (dd, *J* = 6.3 and 9.4 Hz, 1H, one of the CH_2-3 group), 3.98 (dd, *J* = 5.8 and 9.4 Hz, 1H, one of the CH_2-3 group), 4.13 (m, 1H, CH-2), 6.89 (m, 2H, aromatic H-2 and H-6), 6.95 (m, 1H, aromatic H-4), 7.28 (m, 2H, aromatic H-3 and H-5). ^{13}C-NMR (150 MHz, $CDCl_3$) δ (ppm): −4.9 (CH_3-Si), −4.5 (CH_3-Si), 18.1 ($(CH_3)_3\underline{C}$-Si), 25.8 (3C, $(\underline{C}H_3)_3$C-Si), 64.3 (C-1), 68.9 (C-3), 71.2 (C-2), 114.4 (2C, aromatic C-2 and C-6), 120.9 (aromatic C-4), 129.5 (2C, aromatic C-3 and C-5), 158.6 (aromatic C-1). HRMS (ESI): calcd. for $C_{15}H_{26}O_3NaSi$ $[M + Na]^+$ 305.1549; found 305.1545.

2-(*tert*-Butyldimethylosilyloxy)-3-phenoxypropanal (17)

Dess-Martin periodinane (43.79 g, 103.24 mol) was added portionwise to a cold (0 °C) suspension of alcohol **25** (24.3 g, 86.03 mmol) and dry $NaHCO_3$ (21.68 g, 258.09 mmol) in anhydrous CH_2Cl_2 (100 mL). After being stirred for 1 h at room temperature, TLC analysis (AcOEt/hexanes, 1:9) indicated disappearance of the starting alcohol **25**. Saturated aqueous $NaHCO_3$ (350 mL) and Na_2SO_3 (86.72 g, 688.24 mmol) were then added simultaneously and the mixture was stirred at room temperature for 30 min. The resulting layers were separated and the aqueous phase was extracted with CH_2Cl_2 (3 × 50 mL). The combined extracts were washed with water (100 mL), brine (2 × 100 mL) and dried over Na_2SO_4. Filtration and evaporation in vacuo furnished the crude product as a light yellow oil, which was purified by flash column chromatography (silica gel, 2%–10% *tert*-butylmethyl ether/hexanes) to give the aldehyde **17** [5] (22.61 g, 93.7% yield) as a colourless oil. R_f = 0.56 (20% AcOEt/hexanes). FT-IR (thin film) ν (cm^{-1}): 3042, 2954, 2930, 2885, 2858, 1740, 1600, 1589, 1497, 1472, 1463, 1389, 1362, 1302, 1247, 1172, 1143, 1081, 1059, 1006, 975, 939, 838, 811, 781, 753, 691, 671, 509. ^{1}H-NMR (600 MHz, $CDCl_3$) δ (ppm): 0.13 (s, 3H, CH_3-Si), 0.16 (s, 3H, CH_3-Si), 0.93 (s, 9H, $(CH_3)_3$C-Si), 4.07 (dd, *J* = 6.7 and 9.8 Hz, 1H, one of the CH_2-3 group), 4.22 (dd, *J* = 3.8 and 9.8 Hz, 1H, one of the CH_2-3 group), 4.40 (ddd, *J* = 0.8, 3.8 and 6.7 Hz, 1H, CH-2), 6.90 (m, 2H, aromatic H-2 and H-6), 6.97 (m, 1H, aromatic H-4), 7.28 (m, 2H, aromatic H-3 and H-5), 9.75 (d, *J* = 0.8 Hz, 1H, -CHO). ^{13}C-NMR (150 MHz, $CDCl_3$) δ (ppm): −4.9 (CH_3-Si), −4.7 (CH_3-Si), 18.3 ($(CH_3)_3\underline{C}$-Si), 25.7 (3C, $(\underline{C}H_3)_3$C-Si), 68.8 (C-3), 76.9 (C-2), 114.5 (2C, aromatic C-2 and C-6), 121.2 (aromatic C-4), 129.5 (2C, aromatic C-3 and C-5), 158.3 (aromatic C-1), 202.3 (-CHO). HRMS (ESI): calcd. for $C_{15}H_{24}O_3NaSi$ $[M + Na]^+$ 303.1392; found 303.1386.

Abbreviations

AcOEt	ethyl acetate
DIAD	diisopropyl azodicarboxylate
DIBAL-H	diisobutylaluminum hydride
DMF	*N*,*N*-dimethylformamide
DMP	Dess-Martin periodinane
EtOH	ethanol
ImH	imidazole
MeOH	methanol

PivCl	trimethylacetyl chloride
Py	pyridine
TBDMSCl	*tert*-butyldimethylsilyl chloride
TsCl	*p*-toluenesulfonyl chloride
THF	tetrahydrofuran

References

1. Tipson, R.S.; Clapp, M.A.; Cretcher, L.H. Cinchona alkaloids in pneumonia. XI. Some ethers of Apocupreine. *J. Am. Chem. Soc.* **1943**, *65*, 1092–1094.
2. Mohammadpoor-Baltork, I.; Khosropour, A.R.; Aliyan, H. Efficient conversion of epoxides to 1,3-dioxolanes catalyzed by bismuth (III) salts. *Synth. Commun.* **2001**, *31*, 3411–3416.
3. Bredikhin, A.A; Bredikhina, Z.A.; Novikova, V.G.; Pashagin, A.V.; Zakharychev, D.V.; Gubaidullin, A.T. Three different types of chirality-driven crystallization within the series of uniformly substituted phenyl glycerol ethers. *Chirality* **2008**, *20*, 1092–1103.
4. Theil, F.; Lemke, K.; Ballschuh, S.; Kunath, A.; Schick, H. Lipase-catalysed resolution of 3-(aryloxy)-1,2-propanediol derivatives—Towards an improved active site model of *Pseudomonas cepacia* Lipase (Amano PS). *Tetrahedron Asymm.* **1995**, *6*, 1323–1344.
5. Lin, P.; Chang, L.; Edmondson, S.D. Pyrrolidine-Derived Beta 3 Adrenergic Receptor Agonists. WO Patent 2010/129326 A1, 11 November 2010.
